# Managing a Primary Care Pediatric Practice Through COVID-19

**DOI:** 10.1017/dmp.2020.488

**Published:** 2020-12-22

**Authors:** Ellen K. Hamburger, Kandra Strauss-Riggs, Joelle N. Simpson

**Affiliations:** 1 Children’s National Hospital, General and Community Pediatrics, Washington, DC, USA; 2 The National Center for Disaster Medicine and Public Health and the Department of Military & Emergency Medicine, Uniformed Services University of the Health Sciences, Bethesda, Maryland, USA; 3 The Henry M. Jackson Foundation for the Advancement of Military Medicine, Inc., Rockville, Maryland, USA; 4 Children’s National Hospital, Emergency Medicine and Trauma Center, Washington, DC, USA

**Keywords:** Primary prevention, epidemics, pediatric preparedness, COVID-19

The coronavirus disease 2019 (COVID-19) is rapidly spreading in the United States and health-care providers of all types need to continue to provide care for their patients while keeping themselves and their staff safe. While children’s hospitals have been engaged in disaster management training for many years, primary care pediatric practices were unlikely to have an operational plan to manage an infectious disease outbreak to this scale.^1,2^ General pediatric practices typically see relatively healthy patients, presuming that those who are very sick during an infectious disease outbreak will go to the emergency department (ED) for care. In the COVID-19 pandemic, however, children with the disease typically exhibit very mild symptoms that overlap with common viral illnesses and are unlikely to present to the ED. The most commonly reported symptoms in the pediatric population includes fever, cough and shortness of breath.^3–6^ Additionally, the public health and medical community is urging the public to stay away from the EDs to avoid crowding. We have also seen a decline in visits to tertiary care pediatric EDs likely due to local stay at home orders.^7^ They are the ones determining who gets a COVID-19 test, who needs to be referred for more complex care in the hospital, and how to protect their health-care teams and other patients from those who may be spreading the disease in their waiting rooms.

The flood of rapidly evolving information on COVID-19, much of it aimed at adult patients, poses challenges for pediatricians to digest and operationalize in their daily practice.^7–12^ Even in the face of these challenges, office teams are creatively applying new systems to protect their staff and patients. These include using telemedicine; asking families to wait in the car, rather than the waiting room, to receive a text when the doctor can see them; scheduling well patient visits for the morning and sick patients in the afternoon; triaging which routine appointments to re-schedule; or having a plan for how to isolate a suspected COVID-19 patient. Some facilities are moving to drive-through testing and giving routine vaccines to patients in their car seats. Communicating these altered procedures to patients, parents, and staff, and reassuring them that it is for their protection, is an entirely new role for pediatric practices.

It is important to remember the common infrastructure that is already in place. The Centers for Disease Control and Prevention (CDC) and the American Academy of Pediatrics (AAP), among others, have relevant emergency preparedness guidance and enduring materials related to infectious disease management.^13^ The AAP shares guidance on children and disasters including principles of emergency management for pediatricians.^14^ Emergency management refers to the framework within which communities reduce vulnerability to hazards and cope with disasters.^15,16^ This information can be adapted for primary care pediatric practice operations by building on the procedures, structures, and communication tools they already have. For example, many pediatric practices have online portals for their patients and made use of those communication channels to provide COVID-19 updates to their patients.^4–8^

While hospital-based clinicians may have had emergency management experience, such as incident command system (ICS) training to respond to a disaster, primary care pediatricians are learning the principles of emergency management in the midst of a prolonged event, when staff are already stressed. While caring for patients and their families throughout challenging times is part of the pediatrician’s role as a primary care provider, the language of continuity of operations, redundancy of systems, and ICS may be new to this community.^15–17^ It is not realistic for the general primary care pediatrician to learn an entirely new field during the COVID-19 pandemic, but they can certainly apply the lens of emergency management to the day to day care they are providing. The basics of emergency management might involve assigning roles to staff, such as patient communication lead (known in ICS as the Liaison Officer), researcher (Public Information Officer), office manager (Safety Officer), billing (Finance Lead), and practice director (Incident Commander). The owner or medical director of a practice leads every day; it just looks a little different when leveraging skills on their staff to help manage a prolonged crisis like COVID-19.

If a pediatrician does need to suspend their practice, there are ways to continue contributing to the national response through the National Disaster Medical System (NDMS) or Medical Reserve Corps (MRC).^17,18^ These might be opportunities to provide roles for providers and staff, even if they cannot deliver direct patient care in their practice.

The COVID-19 pandemic will eventually ebb—therapeutics, an antiviral, and/or vaccines are being developed. Perhaps one of the lasting outcomes from this challenging time will be that primary care pediatric practices will integrate emergency management principles into their work on a regular basis. Independent practices might consider establishing memorandums of understanding (MOUs) with area children’s hospitals, local health-care coalitions, or their AAP chapter network to quickly tap expertise at that level when an event occurs. Pediatric practices can develop pre-vetted roles within the incident command system that are exercised on a regular basis, so staff are trained and prepared to pivot into that role when an emergency occurs. The basics of emergency management might involve assigning roles to staff such as patient communication lead (known in ICS as the Liaison Officer), researcher (Public Information Officer), office manager (Safety Officer), billing (Finance Lead), and practice director (Incident Commander). The Safety Officer could be tasked with reviewing the AAP pediatric preparedness checklist for practices which highlights planning for implementation of infection control practices or vaccine management. The liaison officer can reach out to partners in the local health-care coalition to collaborate on strategies for mitigation or recovery efforts. The Finance lead should be aware of potential economic injury disaster loans and federal aid programs that can provide relief from any temporary loss of revenue. The owner or medical director of a practice leads every day; it just looks a little different when leveraging skills on their staff to help manage a prolonged crisis like COVID-19.^19^

As we look ahead to documenting the lessons of this pandemic, it would be useful to know how emergency management principles have, or have not, been adopted by primary care pediatricians. Is the incident command system applicable to the primary care pediatric environment? Is it helpful to use these principles when managing a prolonged disaster like COVID-19? Further research into such questions will be critical in assessing our ability to better mitigate, prepare for, respond to and recover from future events. Such inquiry has implications for training health-care professionals and further preparing pediatric practices to manage their patients. It is also critical in clarifying their role in the systemic response to disasters that have profound impact on health outcomes for the pediatric population.
